# Immunomodulatory effects of chicken cathelicidin-2 on a primary hepatic cell co-culture model

**DOI:** 10.1371/journal.pone.0275847

**Published:** 2022-10-10

**Authors:** Csilla Sebők, Stephanie Walmsley, Patrik Tráj, Máté Mackei, Júlia Vörösházi, Janka Petrilla, László Kovács, Ágnes Kemény, Zsuzsanna Neogrády, Gábor Mátis

**Affiliations:** 1 Department of Physiology and Biochemistry, Division of Biochemistry, University of Veterinary Medicine, Budapest, Hungary; 2 Department of Animal Hygiene, Herd Health and Mobile Clinic, University of Veterinary Medicine, Budapest, Hungary; 3 Department of Pharmacology and Pharmacotherapy, Faculty of Medicine, University of Pécs, Pécs, Hungary; University of Michigan Health System, UNITED STATES

## Abstract

Cathelicidin-2 is an antimicrobial peptide (AMP) produced as part of the innate immune system of chickens and might be a new candidate to combat infection and inflammation within the gut-liver axis. Studying the hepatic immune response is of high importance as the liver is primarily exposed to gut-derived pathogen-associated molecular patterns. The aim of the present study was to assess the effects of chicken cathelicidin-2 alone or combined with lipoteichoic acid (LTA) or phorbol myristate acetate (PMA) on cell viability, immune response and redox homeostasis in a primary hepatocyte—non-parenchymal cell co-culture of chicken origin. Both concentrations of cathelicidin-2 decreased the cellular metabolic activity and increased the extracellular lactate dehydrogenase (LDH) activity reflecting reduced membrane integrity. Neither LTA nor PMA affected these parameters, and when combined with LTA, cathelicidin-2 could not influence the LDH activity. Cathelicidin-2 had an increasing effect on the concentration of the proinflammatory CXCLi2 and interferon- (IFN-)γ, and on that of the anti-inflammatory IL-10. Meanwhile, macrophage colony stimulating factor (M-CSF), playing a complex role in inflammation, was diminished by the AMP. LTA elevated IFN-γ and decreased M-CSF levels, while PMA only increased the concentration of M-CSF. Both concentrations of cathelicidin-2 increased the H_2_O_2_ release of the cells, but the concentration of malondialdehyde as a lipid peroxidation marker was not affected. Our findings give evidence that cathelicidin-2 can also possess anti-inflammatory effects, reflected by the alleviation of the LTA-triggered IFN-γ elevation, and by reducing the M-CSF production induced by PMA. Based on the present results, cathelicidin-2 plays a substantial role in modulating the hepatic immune response with a multifaceted mode of action. It was found to have dose-dependent effects on metabolic activity, membrane integrity, and reactive oxygen species production, indicating that using it in excessively high concentrations can contribute to cell damage. In conclusion, cathelicidin-2 seems to be a promising candidate for future immunomodulating drug development with an attempt to reduce the application of antibiotics.

## Introduction

Antimicrobial peptides (AMP) are small, cationic peptides produced by almost all organisms. Although they have been found to serve an antimicrobial role, recent studies discovered that these effects are less pronounced *in vivo*, because the high salt and glycosaminoglycan concentrations can inhibit their effect under physiological conditions [1]. As a novel approach, it can be suggested that the health-promoting properties of these AMPs are mostly based on their immunomodulatory role instead of the direct antimicrobial effects. They help to maintain the balance of inflammatory responses, and therefore support the organism in fighting infections with its own resources [2].

One group of AMPs that have been of great interest recently are cathelicidins. This group of molecules is produced as part of the innate immune system of various species, including humans and birds. They have been proved to be effective against various bacteria, fungi and viruses [3–5], but they are also implicated in signaling cell damage, recruiting and activating immune cells, and promoting wound healing [3]. The mechanism of cathelicidins’ immunomodulatory role is complex and appears to involve the activation of multiple intracellular signaling pathways. Chicken cathelicidin-2 belongs to this group, and there is currently a gap in literature surrounding its immunomodulatory activities in different tissue types. Research efforts focusing on the characterization of these complex interactions would aid in the assessment of cathelicidin-2’s suitability as a therapeutic agent.

Literature data regarding the effects of AMPs on the chicken liver is yet very limited. However, to learn more about the function of the liver would be of great importance in the future, as it plays a key role in the inflammatory response triggered by gastroenteric microorganisms. It is the first organ that comes in contact with pathogen-associated molecular patterns absorbed from the gastrointestinal (GI) system and serves as a filter between the digestive tract and the rest of the body [6, 7]. Receptors on hepatocytes, Kupffer cells, biliary epithelial cells, and sinusoidal endothelial cells recognize these molecules and induce the production of cytokines [8]. These inflammatory processes have adaptive functions, but excess inflammation can have several harmful effects. This is a major issue in livestock production because it can negatively affect animal welfare and decrease productivity [9].

The Gram-positive bacterial cell wall component, lipoteichoic acid (LTA) acts as a toll-like receptor (TLR) agonist in the liver [10], and was proved to induce oxidative burst in chicken heterophil granulocytes [7, 11, 12]. Phorbol myristate acetate (PMA) is a substance which is frequently used in *in vitro* and *in vivo* models to induce inflammation, triggering pro-inflammatory cytokine release and oxidative stress, although, via a different pathway [13–15]. In our previous study, both LTA and PMA were successfully used to provoke inflammation in 2D and 3D chicken hepatocyte—non-parenchymal cell cultures, reflected by elevated pro-inflammatory cytokine levels, making them suitable for *in vitro* studies related to potential novel anti-inflammatory therapies [16].

Cathelicidins have a diverse effect on inflammation as they can modulate signal pathways and are able to modify the expression of numerous inflammation-related genes [17]. Research studies concerning the effects of cathelicidins on cytokine levels show mixed results. For example, one study found that cathelicidin-2 inhibited the production of tumor necrosis factor-α (TNFα), interleukin (IL)-6, IL-8 and IL-10 by human PBMCs in response to lipopolysaccharide (LPS) exposure but increased the production of Monocyte Chemotactic Protein 1 (MCP-1) [18]. Further, cathelicidin-2 induced transcription of the chemokines CXCLi2/IL-8, MCP-3, and CCLi4/RANTES in a chicken HD11 macrophage cell line [19]. Another AMP, epinecidin-1, was found to modulate cytokine production in lymphocytes of the spleen in mice by stimulating IL-12, IL-4, and IL-10 release without affecting TNF-α production [20]. These data indicate that the role of cathelicidins and other AMPs is not purely anti-inflammatory, but rather immunomodulatory and their effects may differ in various cell types and species. This highlights the importance of characterizing the immunomodulatory activity of chicken cathelicidin-2 in different tissue types if this AMP is to be considered for therapeutic use.

The relationship between the redox homeostasis and inflammation is complex as oxidative stress initiates the activation of certain inflammatory pathways, contributing to the release of more ROS (reactive oxygen species) and RNS (reactive nitrogen species) from immune cells such as neutrophils and macrophages [21–23]. The production of ROS can result in lipid peroxidation and oxidative damage of proteins, possibly leading to chronic deterioration of the cells [24]. For this reason, combining investigations on the effects of antimicrobial peptides on oxidative stress and inflammation in liver cells is essential to understanding their mechanisms of action.

The aim of the present study was to assess the modulatory role of chicken cathelicidin-2 on the immune response of the liver using a primary hepatic cell co-culture of chicken origin. It would be of great importance to elucidate how this molecule acts on the liver and how it affects inflammatory pathways, as it might be a possible candidate for entering future clinical drug development. For this reason, it should be carefully monitored how different cathelicidin-2 levels can influence cell viability and the hepatocellular redox and inflammatory homeostasis.

## Materials and methods

### Isolation of cells

The liver of a male Ross-308 broiler chicken (*Gallus gallus domesticus*) from Gallus Ltd. (Devecser, Hungary) was used for the isolation of liver cells. Feeding was carried out according to Ross Technology [25] and the chicken was slaughtered at three weeks of age. All experiments were in accordance with European Union laws, institutional guidelines, confirmed by the Local Animal Welfare Committee of the University of Veterinary Medicine Budapest, and permitted by the Government Office (number of permission: GK-419/2020; approval date: 11 May 2020). All efforts were made to minimize suffering of the animal. Unless stated otherwise, all chemicals were purchased from Sigma-Aldrich (Darmstadt, Germany).

To collect the liver for cell isolation, slaughter of the chicken was done under carbon dioxide narcosis by decapitation. The gastropancreaticoduodenal vein was cannulated, then a three-step perfusion was performed with buffers containing ethylene glycol tetraacetic acid (EGTA) and Hanks’ Balanced Salt Solution Buffer (HBSS), as described by Mackei et al. [26]. In the final step of the perfusion the buffers were supplemented with 100 mg collagenase type IV (Nordmark, Uetersen, Germany). After the liver was removed and the Glisson’s capsule was disrupted, the cells were suspended in 50 mL ice cold bovine serum albumin (BSA) containing HBSS buffer and filtered through three layers of sterile gauze. This cell suspension was then incubated on ice for 45 minutes to avoid the aggregation of cells, then the suspension was centrifuged three times at 100x g for 3 minutes. The pellet containing hepatocytes was resuspended in Williams Medium E supplemented with 0.22% NaHCO_3_, 50 mg/mL gentamycin, 0.5 μg/mL amphotericin B, 2 mM glutamine, 4 μg/L dexamethasone, 20 IU/L insulin, and 5% fetal bovine serum (FBS). The medium contained the FBS only in the first 24 hours after seeding.

The supernatant containing non-parenchymal cells was centrifuged again at 350x g for 10 minutes to ensure sedimentation of residual hepatocytes, cellular debris, and erythrocytes. After this centrifugation, the supernatant was collected and centrifuged at 800x g for 10 minutes, then the pellet comprised of hepatic non-parenchymal cells was resuspended in Williams Medium E. Trypan blue exclusion test was applied to assess the viability of hepatocyte and non-parenchymal cell fractions, and over 90% of the cells were found to be viable. A Bürker’s chamber was used to count cell numbers to ensure appropriate cell concentrations in cultures. The cell suspensions were diluted to 8.5*10^4^ cells/mL in the hepatocyte-enriched fraction and to 1.5*10^5^ cells/mL in the non-parenchymal cell-containing fraction. Hepatocyte and liver non-parenchymal cell fractions were previously characterized with flow cytometry and immunofluorescent detection of hepatocyte and macrophage-specific markers [26].

### Preparing cell cultures

24-well and 96-well (Greiner Bio-One, Frickenhausen, Germany) culture plates coated with collagen type I were used to prepare cell cultures. Before plating, the hepatocyte and the non-parenchymal cell suspensions were mixed in 6:1 ratio. Seeding volumes were 400 μL per well on the 24-well plates and 100 μL per well on the 96-well plates. All cell cultures were incubated at 37°C with high humidity and 5% CO_2_, and culture medium was changed after 4 hours. 24 hours of incubation yielded confluent monolayers.

### Treatments

After the 24-hour incubation time of seeded cell cultures, the media was removed and replaced by culture media containing the investigated agents. Cells were treated with chicken cathelicidin-2 in 5 nmol/mL and 10 nmol/mL concentrations (Isca Biochemicals, Exeter, Devon, UK). *Staphylococcus aureus*-derived LTA (Sigma-Aldrich, Darmstadt, Germany) was used in 50 μg/ml, and PMA (Sigma-Aldrich, Darmstadt, Germany) in 1000 ng/mL concentrations. The treatment groups are presented in Table 1 (n = 6/group). After another 24 hours of incubation, samples were taken from the cell culture media and stored at -80°C until further measurements.

**Table 1:** Treatment groups.

Group	Cathelicidin-2	LTA	PMA
Control	-	-	-
Cath-5	5 nmol/mL	-	-
Cath-10	10 nmol/mL	-	-
LTA	-	50 μg/mL	-
LTA+Cath-5	5 nmol/mL	50 μg/mL	-
PMA	-	-	1000 ng/mL
PMA+Cath-5	5 nmol/mL	-	1000 ng/mL

### Measurements

To assess the metabolic activity of the cultured cells, CCK-8 assay was used on 96-well plates to detect the amount of NADH+H^+^ produced in the catabolic pathways. The assay contains a water soluble tetrazolium salt (WST-8) which is reduced by cellular dehydrogenase enzymes producing a yellow-color formazan dye, which is soluble in the culture medium. The amount of the formazan dye, generated by the dehydrogenases of the cells, refers to the cellular catabolic metabolic activity. The test was performed following the manufacturer’s directions. 10 μL of CCK-8 reagent and 100 μL of fresh Williams Medium E were added to each well of the 96-well plate, and it was incubated at 37°C for 2 hours. Absorbance was measured at 450 nm with a Multiskan GO 3.2 reader (Thermo Fisher Scientific, Waltham, MA, USA).

Lactate dehydrogenase (LDH) activity was measured with an enzyme kinetic photometric assay (Diagnosticum Ltd., Budapest, Hungary) from the cell culture medium of cells on 24-well plates to assess membrane damage. 200 μL of the working reagent (56 mM phosphate buffer, pH = 7.5, 1.6 mM pyruvate, 240 μM NADH+H^+^) was added to each well of a microplate, along with 10 μL of samples. Absorbance was measured at 340 nm with a Multiskan GO 3.2 reader. The LDH activity was calculated by measuring the absorbance six times in one-minute periods while incubating the mixture at 37°C, and averaging the differences between the consecutive time points.

CXCLi2 concentrations were measured in the culture media of the 24-well plates with chicken-specific ELISA kits (MyBioSource, San Diego, CA, USA) following the manufacturer’s instructions. Absorbance values were obtained with a Multiskan GO 3.2 reader at 450 nm.

Luminex xMAP technology was used to determine the protein concentrations of IFN-γ, IL-10, and M-CSF, performing Milliplex Chicken Cytokine/Chemokine Panel (Cat.Nr.: GCYT1-16K, Merck KGaA, Darmstadt, Germany) according to the instructions of the manufacturer. Briefly, all samples were thawed and tested in a blind-fashion and in duplicate. 25 ml volume of each sample, standard, control, and assay buffer was added to a 96-well plate (provided with the kit). An additional 25 μl of five, distinctly colored, capture antibody-coated bead sets were added to each well. After overnight incubation, biotinylated detection antibody mixture and streptavidin phycoerythrin were added to the plate following appropriate washing steps. After the last washing step, 150 ml drive fluid was added to the wells, the beads were resuspended for an additional 5 minutes on a plate shaker and read on the Luminex MAGPIX® instrument. Luminex xPonent 4.2 software was used for data acquisition. Five-PL regression curves were generated to plot the standard curves for all analytes by the Milliplex Analyst 5.1 (Merck Millipore, Darmstadt, Germany) software calculating with bead median fluorescence intensity (MFI) values.

The fluorometric Amplex Red method (Thermo Fisher Scientific, Waltham, MA, USA) was used to detect extracellular H_2_O_2_ content in the culture medium. A Victor X2 2030 fluorometer (Perkin Elmer, Waltham, MA, USA) was used to detect fluorescence (λex = 560 nm, λem = 590 nm) after a 30-minute incubation of 50 μL freshly prepared Amplex Red (100 μM) and HRP (0.2 U/mL) containing working solution with 50 μL culture medium at room temperature (21°C).

A specific colorimetric assay was used to detect malondialdehyde (MDA) concentration as a marker of lipid peroxidation in cell culture medium. 300 μL of freshly prepared thiobarbituric acid stock solution was mixed with 100  μL cell culture medium according to the protocol. After 1 hour of incubation at 95°C, the solutions were cooled on ice for 10 minutes. A Multiskan GO 3.2 reader was used to measure absorbance at 532 nm.

### Statistical analysis

All statistical analyses were performed in R v. 4.0.3 (R Core Team, 2020). By dividing each value by the average of the relevant control group, relative values were derived from the data for visualization. Plots were generated using the ggplot2 package [27]. During statistical analysis, each treatment was compared to its corresponding control group. All groups were compared to Control, the LTA+Cath-5 to the LTA group, and the PMA+Cath-5 to the PMA group. Significance of the differences was evaluated by two-sample t-tests. We have considered a difference statistically significant if the p was less than 0.05.

## Results

### Cellular metabolic activity

**Fig 1:**
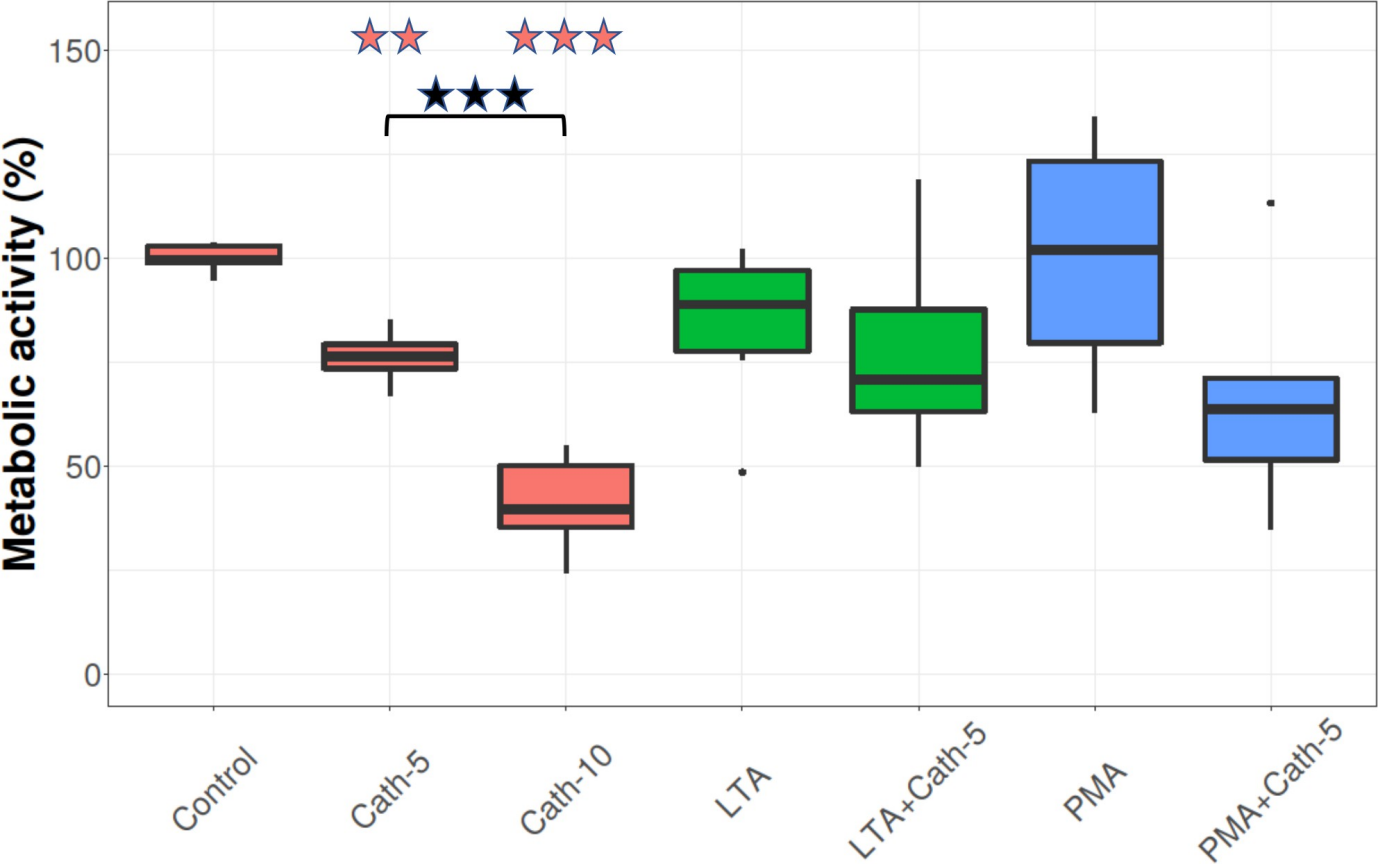
Metabolic activity. Boxplots showing the metabolic activity for hepatocyte–non-parenchymal cell co-cultures treated with chicken cathelicidin-2 (Cath), lipoteichoic acid (LTA), or phorbol myristate acetate (PMA) only, or the combination of Cath and LTA or PMA (n = 6/group). Results are displayed as percentage, where 100% is the mean value of control cultures. Cath-5 = 5 nmol/mL Cath, Cath-10 = 10 nmol/mL Cath, LTA = 50 μg/mL *Staphylococcus aureus* LTA, PMA = 1000 ng/mL PMA. The Control refers to absolute control cells that received none of the treatments. Red asterisks over the boxes refer to significant differences compared to control cells, green asterisks to the group treated only with LTA, blue asterisks to the group treated only with PMA, and black asterisks between the two concentrations of Cath. ** p < 0.01, *** p < 0.001.

Metabolic activity was significantly decreased in the samples treated with both concentrations of cathelicidin-2 (Cath-5 and Cath-10) compared to the control, by 23,72% and 58,97%, respectively, as seen in **Fig 1** (p = 0.004, p < 0.001, respectively), The difference between the decrease caused by the two concentrations of the peptide were significant (p < 0.001). LTA or PMA exposure did not affect significantly the metabolic activity of the cells when compared to the control group. The cells treated with PMA and Cath-5 together showed decreased metabolic activity compared to control (p = 0.026), but this was not true with the LTA+Cath-5 group.

### Lactate dehydrogenase activity

**Fig 2:**
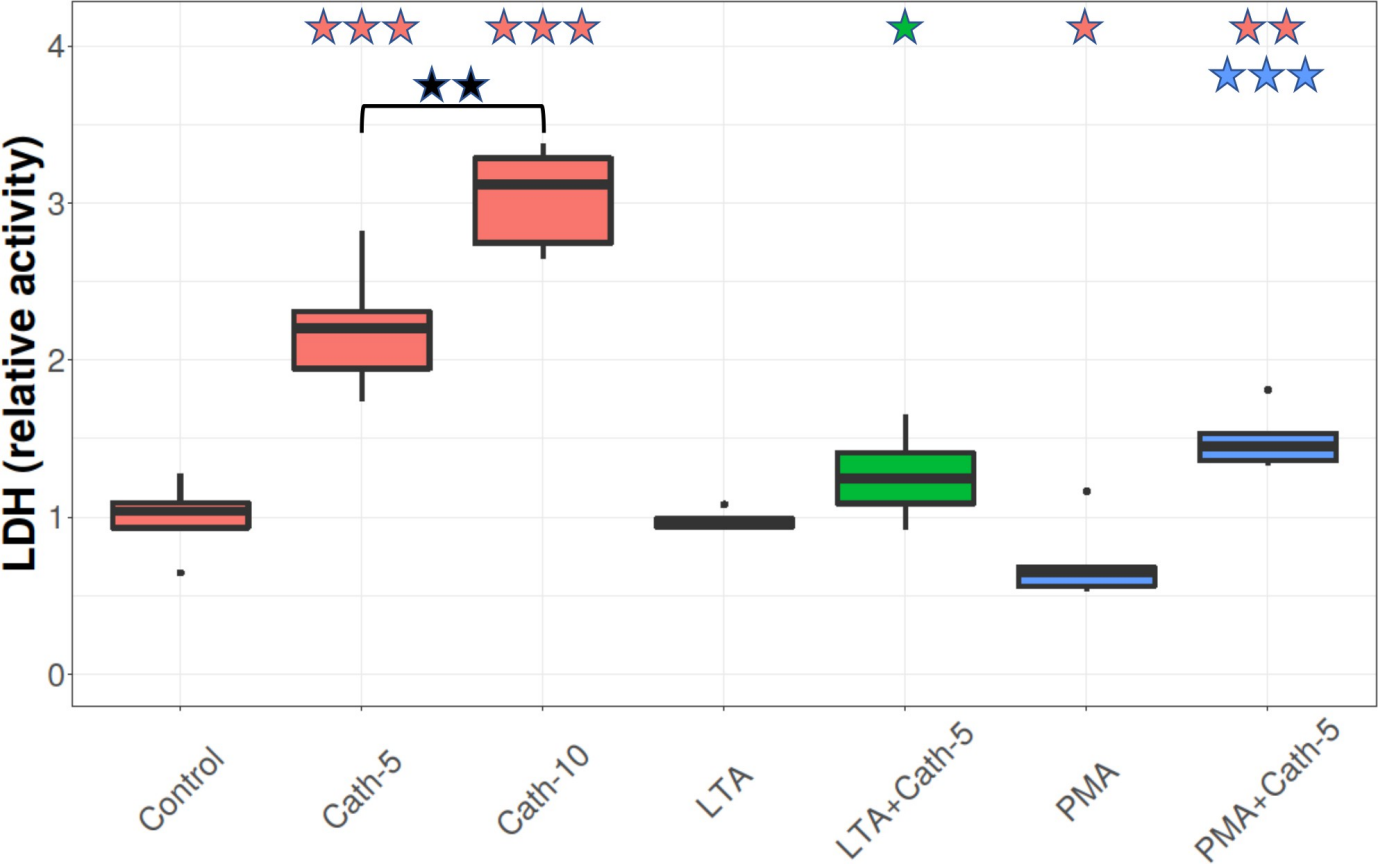
LDH activity. Boxplots showing the lactate dehydrogenase activity for hepatocyte—non-parenchymal cell co-cultures treated with chicken cathelicidin-2 (Cath), lipoteichoic acid (LTA), or phorbol myristate acetate (PMA) only, or the combination of Cath and LTA or PMA (n = 6/group). Results are displayed as relative activity, where 1 is the mean value of control cultures. Cath-5 = 5 nmol/mL Cath, Cath-10 = 10 nmol/mL Cath, LTA = 50 μg/mL *Staphylococcus aureus* LTA, PMA = 1000 ng/mL PMA. The Control refers to absolute control cells that received none of the treatments. Red asterisks over the boxes refer to significant differences compared to control cells, green asterisks to the group treated only with LTA, blue asterisks to the group treated only with PMA, and black asterisks between the two concentrations of Cath. *p< 0.05, *** p < 0.001.

The extracellular LDH activities were increased in the medium of the cells treated with both 5 nmol/mL and 10 nmol/mL cathelicidin-2 (Cath-5 and Cath-10) compared to control cells, (p < 0.001 for both cases), and there was a significant difference between the two concentrations (p = 0.002) (**Fig 2**). LDH activities in the cells that were exposed to LTA showed no significant change compared to the control cells, while PMA slightly decreased the LDH activity (p = 0.045). With regards to the LTA-exposed cells that were treated with cathelicidin-2, the treatment with the lower dose of cathelicidin-2 (LTA+Cath-5) showed a significant increase in LDH levels when compared to the LTA-only condition (p = 0.045), although it did not differ from Control. Cathelicidin-2 supplementation (PMA+Cath-5) increased the LDH activity compared to the group that was treated only with PMA (p < 0.001 in both cases; **Fig 2**), and also compared to Control (p = 0.002).

### IFN-γ concentration

**Fig 3:**
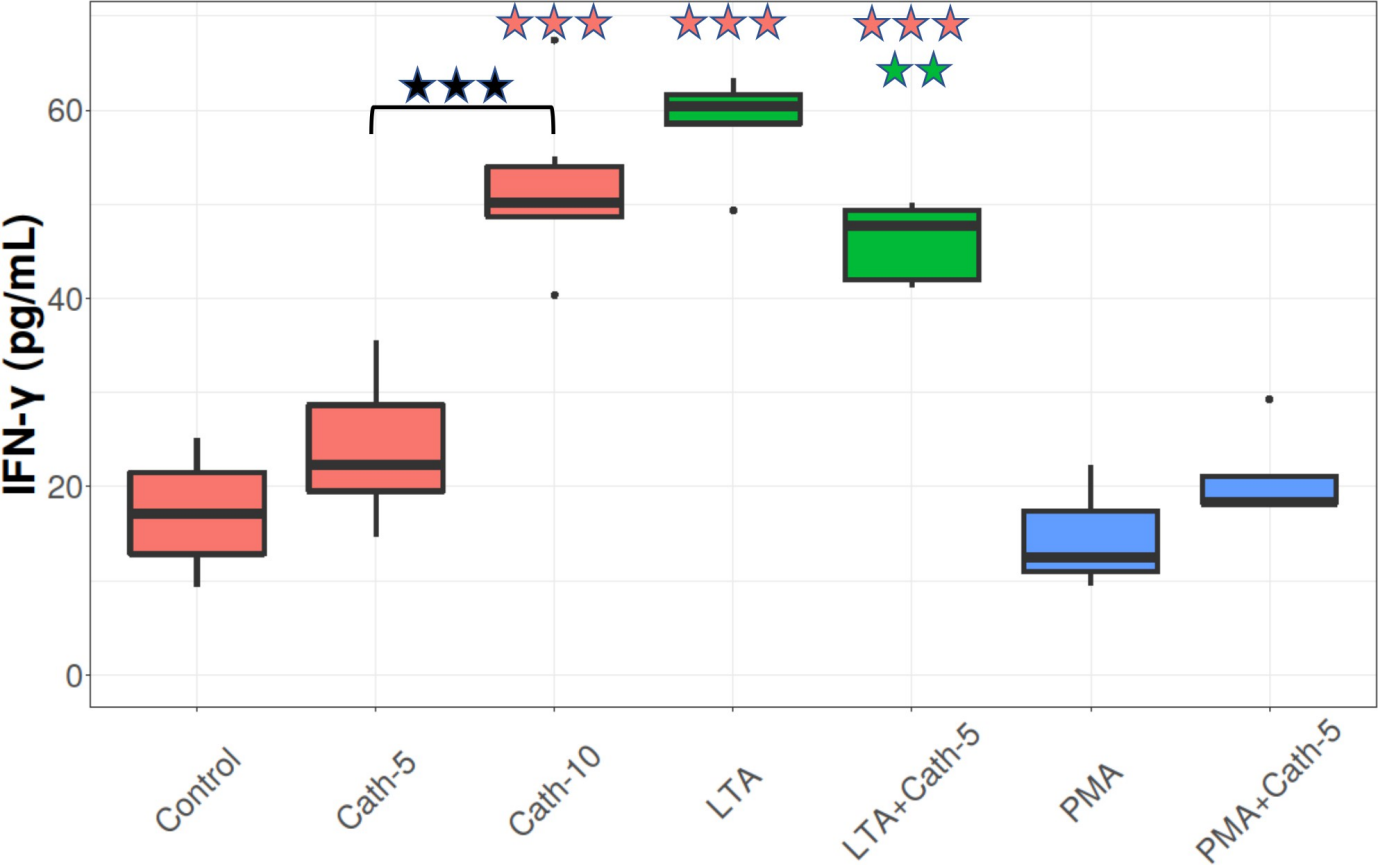
IFN-γ concentration. Boxplots showing the interferon-γ (IFN-γ) concentration for hepatocyte—non-parenchymal cell co-cultures treated with chicken cathelicidin-2 (Cath), lipoteichoic acid (LTA), or phorbol myristate acetate (PMA) only, or the combination of Cath and LTA or PMA (n = 6/group). Results are displayed as pg/mL. Cath-5 = 5 nmol/mL Cath, Cath-10 = 10 nmol/mL Cath, LTA = 50 μg/mL *Staphylococcus aureus* LTA, PMA = 1000 ng/mL PMA. The Control refers to absolute control cells that received none of the treatments. Red asterisks over the boxes refer to significant differences compared to control cells, green asterisks to the group treated only with LTA, blue asterisks to the group treated only with PMA, and black asterisks between the two concentrations of Cath. ** p < 0.01, *** p < 0.001.

There was no significant elevation in IFN-γ levels when treated with the lower concentration of cathelicidin-2 (Cath-5), however, as seen in **Fig 3**, cells treated with the higher dose of cathelicidin-2 (Cath-10) showed higher levels of IFN-γ than the control condition (p < 0.001). The two concentrations showed a significant difference between each other (p < 0.001). Compared to control, the cells exposed to LTA showed higher levels of IFN-γ (p < 0.001). The LTA-exposed cells treated with the lower dose of cathelicidin-2 (LTA+Cath-5) showed a decrease in IFN-γ levels in comparison with the LTA-only condition (p = 0.001), however, the concentration was still higher then in the control cells (p < 0.001). PMA did not affect the IFN-γ production of the cells (**Fig 3**).

### CXCLi2 concentration

**Fig 4:**
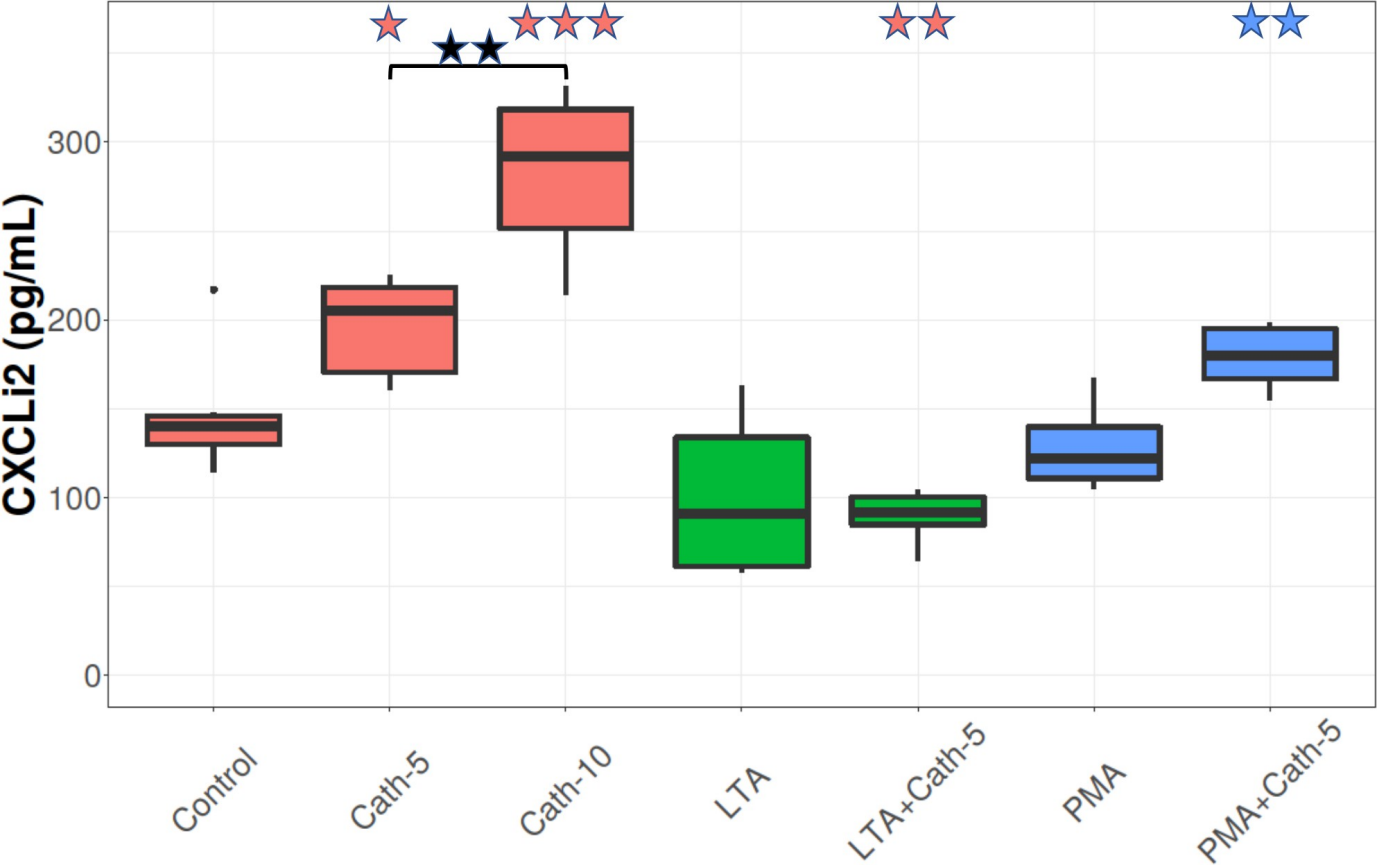
CXCLi2 concentration. Boxplots showing the CXCLi2 concentration for hepatocyte—non-parenchymal cell co-cultures treated with chicken cathelicidin-2 (Cath), lipoteichoic acid (LTA), or phorbol myristate acetate (PMA) only, or the combination of Cath and LTA or PMA (n = 6/group). Results are displayed as pg/mL. Cath-5 = 5 nmol/mL Cath, Cath-10 = 10 nmol/mL Cath, LTA = 50 μg/mL *Staphylococcus aureus* LTA, PMA = 1000 ng/mL PMA. The Control refers to absolute control cells that received none of the treatments. Red asterisks over the boxes refer to significant differences compared to control cells, green asterisks to the group treated only with LTA, blue asterisks to the group treated only with PMA, and black asterisks between the two concentrations of Cath. *p< 0.05, ** p < 0.01, *** p < 0.001.

**Fig 4** represents that cells treated with the lower (Cath-5) and the higher dose (Cath-10) of cathelicidin-2 had significantly increased levels of CXCLi2 compared to the control (p = 0.028, p < 0.001, respectively), and there was a difference between Cath-5 and Cath-10 (p = 0.005). LTA or PMA did not significantly alter the CXCLi2 levels of the cells. In the LTA+Cath-5 group, the CXCLi2 concentrations did not differ from the LTA only group significantly, but there was a difference from Control (p = 0.009). However, in the PMA+Cath-5 group, the CXCLi2 levels were significantly elevated compared to the group that only received PMA (p = 0.002; **Fig 4**).

### IL-10 concentration

**Fig 5:**
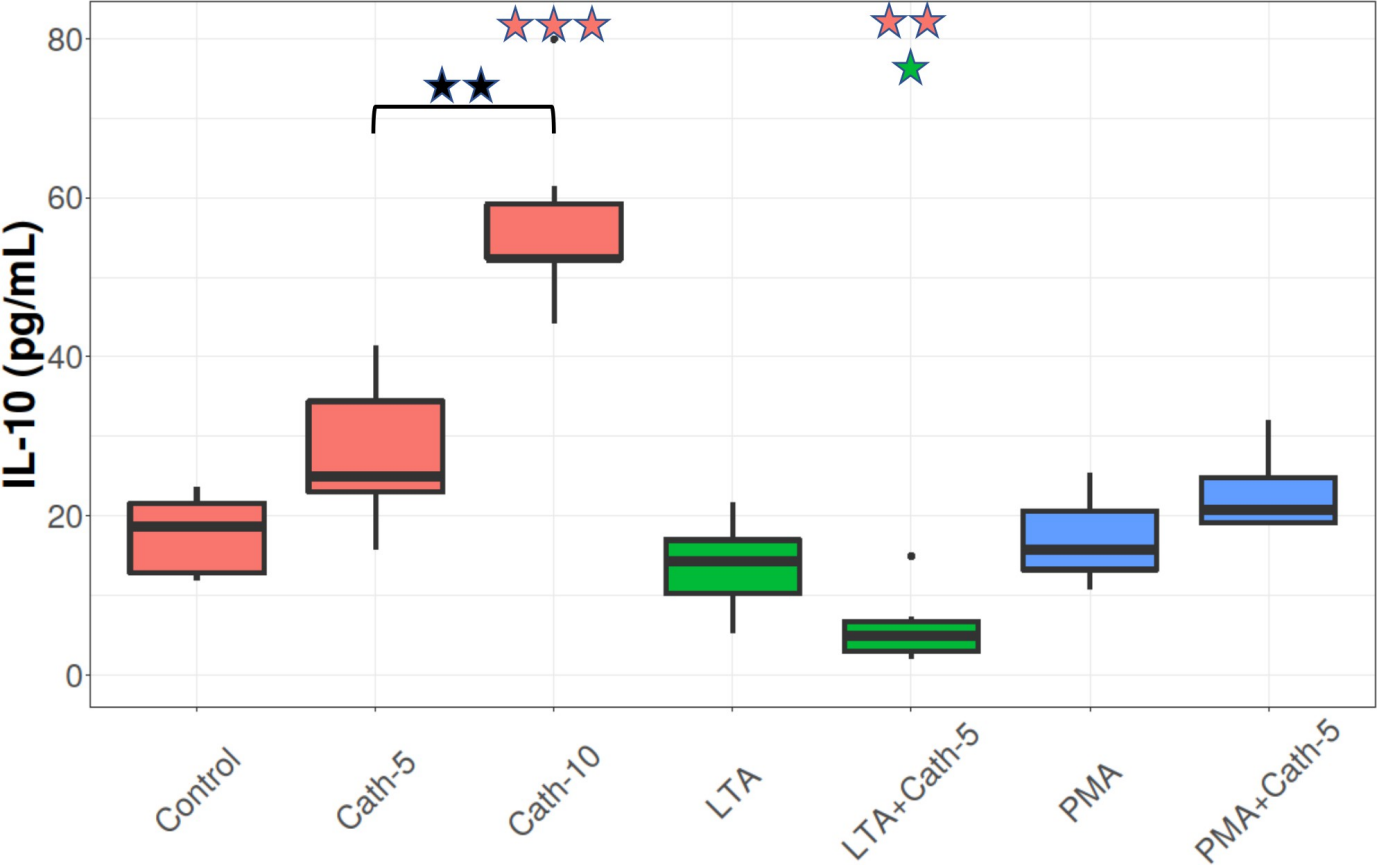
IL-10 concentration. Boxplots showing the interleukin-10 (IL-10) concentration for hepatocyte—non-parenchymal cell co-cultures treated with chicken cathelicidin-2 (Cath), lipoteichoic acid (LTA), or phorbol myristate acetate (PMA) only, or the combination of Cath and LTA or PMA (n = 6/group). Results are displayed as pg/mL Cath-5 = 5 nmol/mL Cath, Cath-10 = 10 nmol/mL Cath, LTA = 50 μg/mL *Staphylococcus aureus* LTA, PMA = 1000 ng/mL PMA. The Control refers to absolute control cells that received none of the treatments. Red asterisks over the boxes refer to significant differences compared to control cells, green asterisks to the group treated only with LTA, blue asterisks to the group treated only with PMA, and black asterisks between the two concentrations of Cath. *p< 0.05, ** p < 0.01, *** p < 0.001.

IL-10 cytokine levels followed patterns mostly similar to CXCLi2 (**Fig 5**). Compared to the control condition, cells treated with only the higher concentration of cathelicidin-2 (Cath-10) showed an increase in IL-10 levels (p < 0.001), so there was a difference between the two concentrations (p = 0.001). There were no significant differences in IL-10 levels between the control and the cell cultures exposed to only LTA or PMA. However, a significant decrease in IL-10 levels was observed in the LTA-exposed cells that were treated with the lower dose of cathelicidin-2 (LTA+Cath-5) compared to the LTA only group (p = 0.033), and it was also higher than the Control group (p = 0.004; **Fig 5**).

### M-CSF concentration

**Fig 6:**
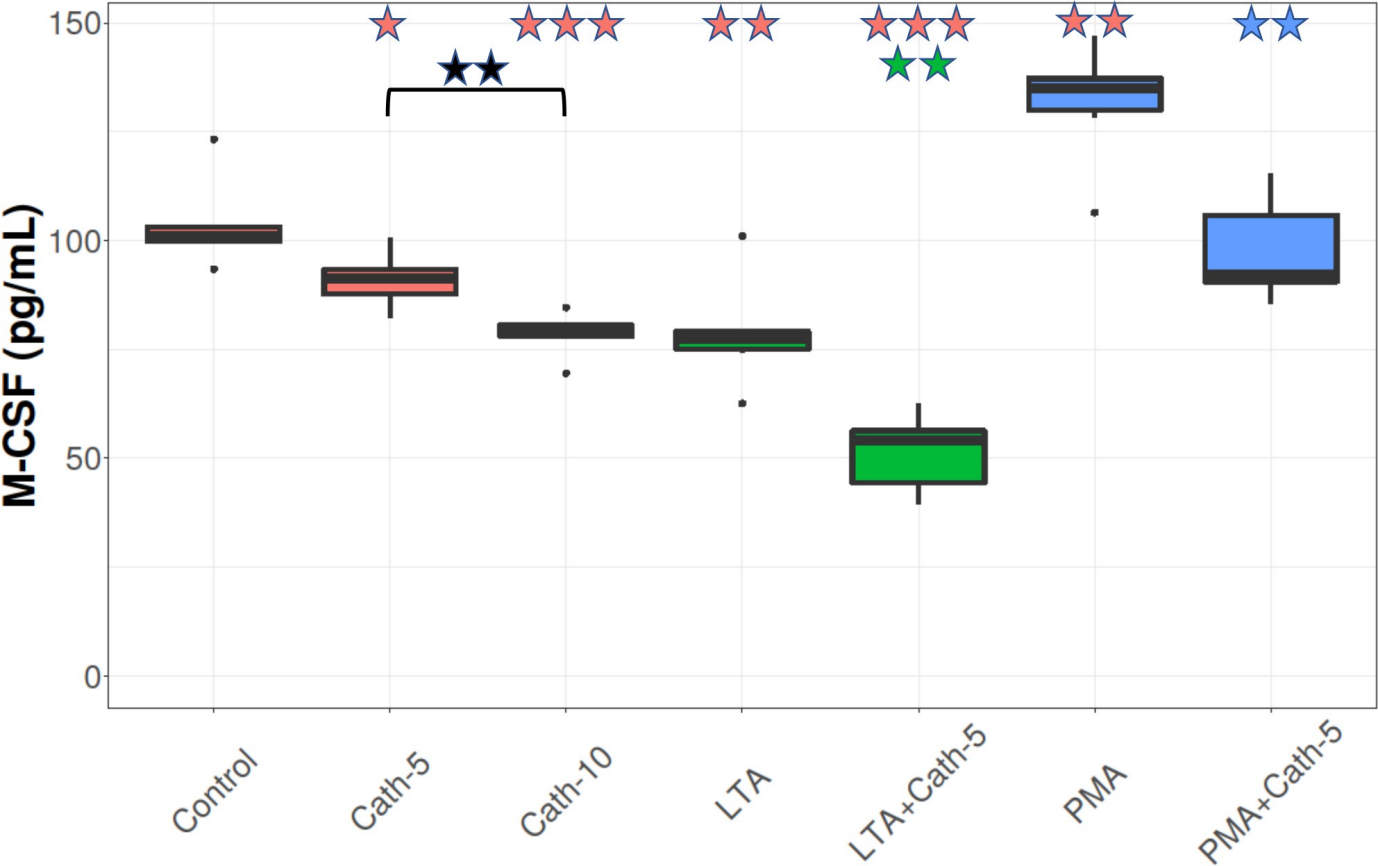
M-CSF concentration. Boxplots showing the macrophage colony stimulating factor (M-CSF) concentration for hepatocyte—non-parenchymal cell co-cultures treated with chicken cathelicidin-2 (Cath), lipoteichoic acid (LTA), or phorbol myristate acetate (PMA) only, or the combination of Cath and LTA or PMA (n = 6/group Results are displayed as pg/mL. Cath-5 = 5 nmol/mL Cath, Cath-10 = 10 nmol/mL Cath, LTA = 50 μg/mL *Staphylococcus aureus* LTA, PMA = 1000 ng/mL PMA. The Control refers to absolute control cells that received none of the treatments. Red asterisks over the boxes refer to significant differences compared to control cells, green asterisks to the group treated only with LTA, blue asterisks to the group treated only with PMA, and black asterisks between the two concentrations of Cath. *p< 0.05, ** p < 0.01, *** p < 0.001.

As seen in **Fig 6**, both cathelicidin-2 concentrations (Cath-5 and Cath-10) significantly decreased the levels of M-CSF (p = 0.031, p < 0.001, respectively) compared to the control group, and there was a difference between the two concentrations (p = 0.004). LTA also showed a depressing effect on the concentrations of this molecule (p = 0.004) compared to the control, and cathelicidin-2 (LTA+Cath-5) further decreased the levels of M-CSF compared to the group that only received LTA (p = 0.003), and it was also lower than Control (p < 0.001). There was a significant increase in the PMA-treated cells (p = 0.003) compared to the absolute control, and in comparison with this group, cathelicidin-2 (PMA+Cath-5) decreased the concentration of M-CSF (p = 0.001), but it did not differ from Control (**Fig 6**).

### H_2_O_2_ concentration

**Fig 7:**
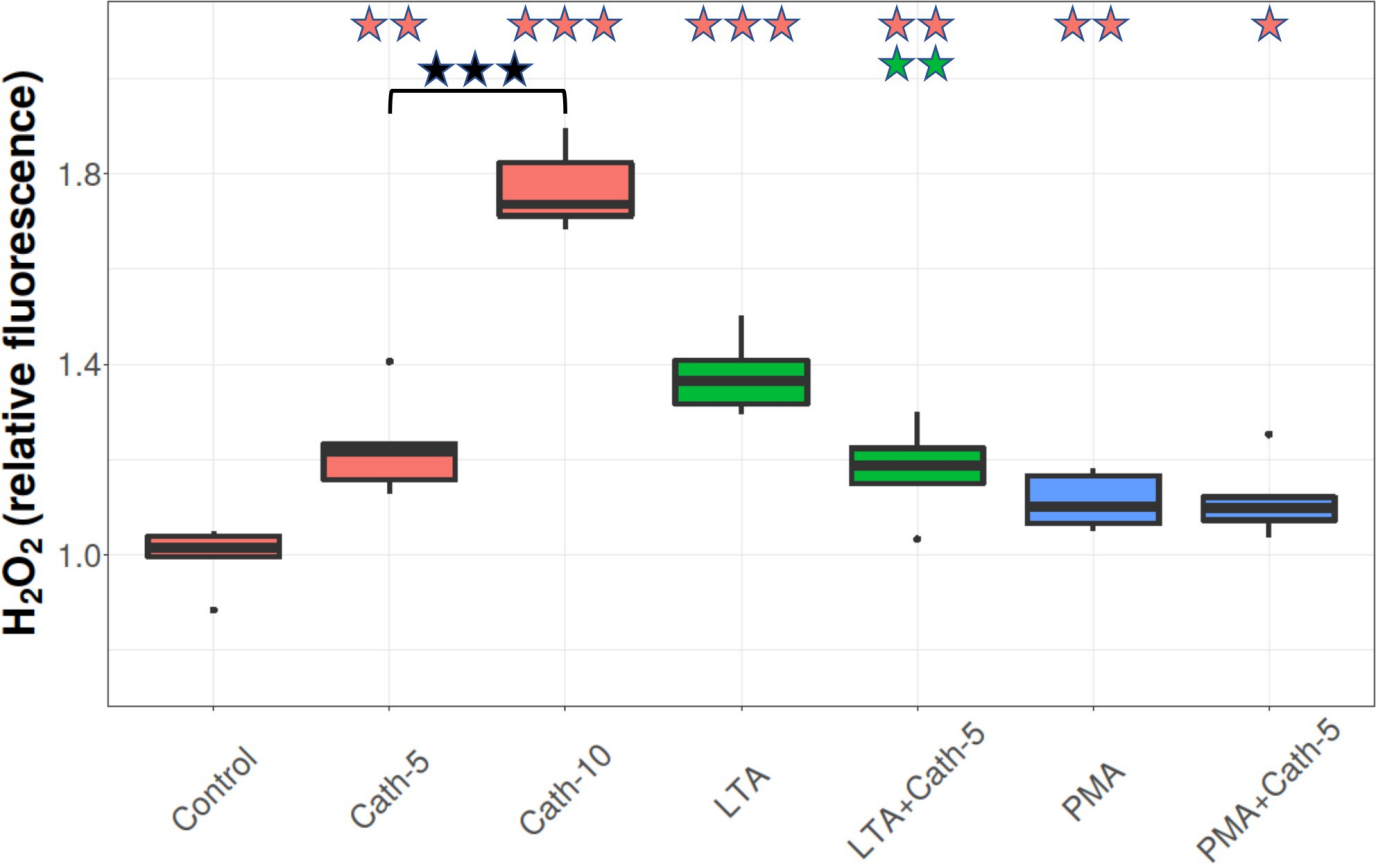
H_2_O_2_ concentration. Boxplots showing the hydrogen peroxide (H_2_O_2_) concentration for hepatocyte—non-parenchymal cell co-cultures treated with chicken cathelicidin-2 (Cath), lipoteichoic acid (LTA), or phorbol myristate acetate (PMA) only, or the combination of Cath and LTA or PMA (n = 6/group). Results are displayed as relative flurescence, where 1 is the mean value of control cultures. Cath-5 = 5 nmol/mL Cath, Cath-10 = 10 nmol/mL Cath, LTA = 50 μg/mL *Staphylococcus aureus* LTA, PMA = 1000 ng/mL PMA. The Control refers to absolute control cells that received none of the treatments. Red asterisks over the boxes refer to significant differences compared to control cells, green asterisks to the group treated only with LTA, blue asterisks to the group treated only with PMA, and black asterisks between the two concentrations of Cath. ** p < 0.01, *** p < 0.001.

The H_2_O_2_ concentrations were elevated after both cathelicidin-2 concentrations (Cath-5 and Cath-10) (p = 0.001, p < 0.001, respectively), and there was a difference between the two concentrations (p< 0.001). LTA and PMA also significantly increased the H_2_O_2_ concentrations (p < 0.001, p = 0.009, respectively), and the lower concentration of cathelicidin-2 (LTA+Cath-5) decreased it compared to the LTA only group (p = 0.002), and compared to Control group (p = 0.003; Fig 7).

### MDA concentration

**Fig 8:**
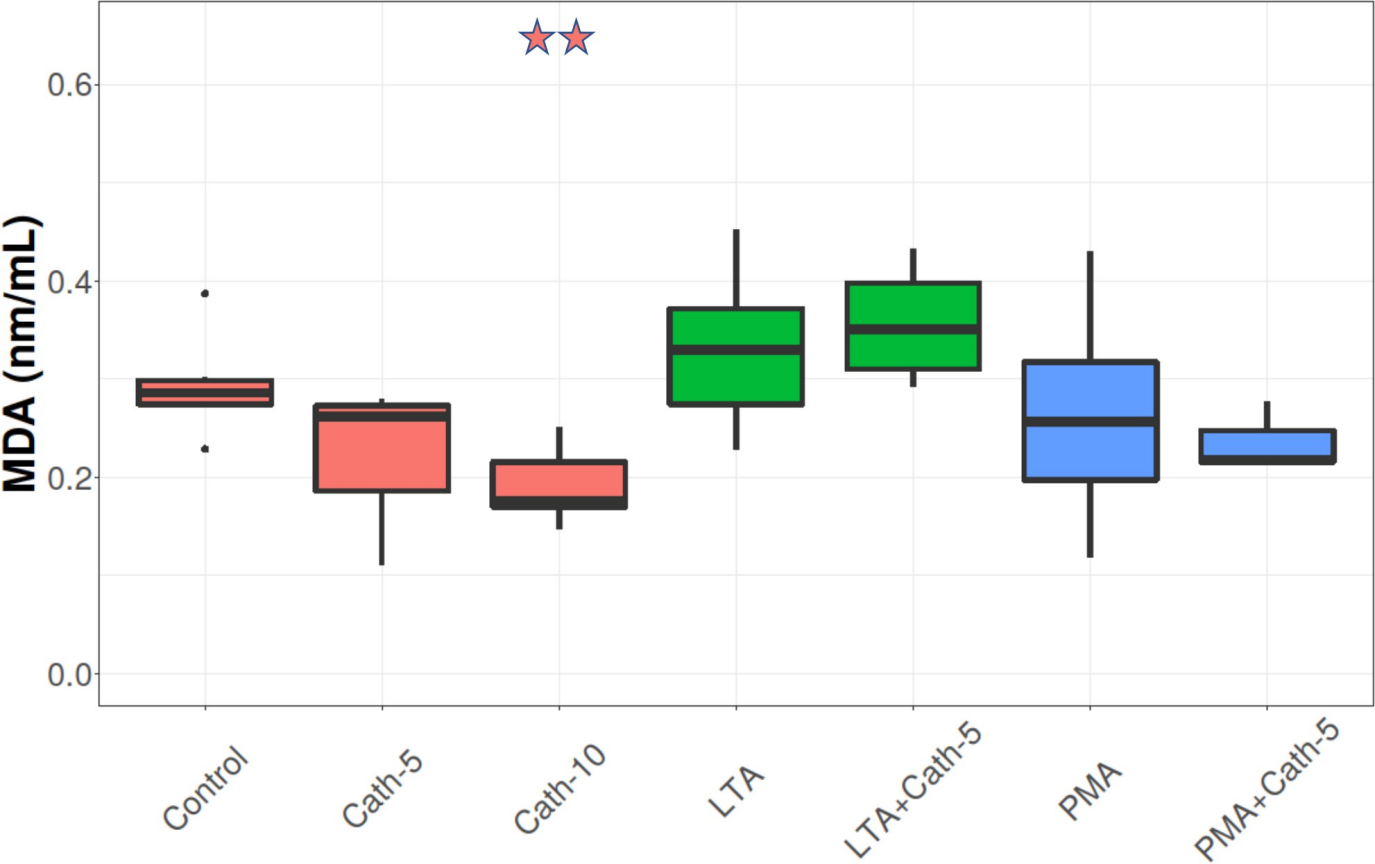
MDA concentration. Boxplots showing the malondialdehyde (MDA) concentration for hepatocyte—non-parenchymal cell co-cultures treated with chicken cathelicidin-2 (Cath), lipoteichoic acid (LTA), or phorbol myristate acetate (PMA) only, or the combination of Cath and LTA or PMA (n = 6/group). Results are displayed as nmol/mL. Cath-5 = 5 nmol/mL Cath, Cath-10 = 10 nmol/mL Cath, LTA = 50 μg/mL *Staphylococcus aureus* LTA, PMA = 1000 ng/mL PMA. The Control refers to absolute control cells that received none of the treatments. Red asterisks over the boxes refer to significant differences compared to control cells, green asterisks to the group treated only with LTA, blue asterisks to the group treated only with PMA, and black asterisks between the two concentrations of Cath. *p< 0.05, ** p < 0.01.

The higher dose of cathelicidin-2 (Cath-10) decreased the concentration of MDA (p = 0.006). Neither LTA, nor PMA changed the MDA levels significantly (**Fig 8**).

## Discussion

In the present study, the effects of the antimicrobial peptide cathelicidin-2 were investigated on a hepatocyte–non-parenchymal cell co-culture of chicken origin, which was previously characterized by our research group [26]. The first question was whether cathelicidin-2 affects the viability and membrane integrity of chicken hepatic cells, and how their inflammatory state is getting altered. These questions should be of great importance, because cathelicidin–as well as other antimicrobial peptides–may have a good chance to enter clinical trials, if the *in vitro* results seem to be promising.

In our study, the metabolic activity and the membrane integrity of the cells were assessed to determine whether cathelicidin-2 has any unfavorable effects on the host cells. It was found that the AMP decreased the metabolic activity and at the same time, increased the LDH activity in cell culture media. These changes were seemingly more considerable with the higher dose of the peptide. However, determining the dose-dependent effect would require testing more concentrations, the lack of which is a limitation of our research. According to our results, the metabolic activity was decreased by approximately 60% with the higher dose of cathelicidin-2, which could indicate remarkable cell damage, but with the lower dose, the decrease was only about 20%, which is considered as just a mild metabolic depression [19, 28]. For this reason, we decided to combine only the lower concentration of cathelicidin-2 with LTA and PMA, as the higher concentration proved to be cytotoxic on our cell culture model. Notwithstanding that cathelicidins bind preferentially to microbial membranes, excess concentrations of these AMPs can result in off-target host cell membrane damage, contributing to LDH release from the cytoplasm and decreased metabolic activity [3, 28].

Neither LTA nor PMA decreased the metabolic activity or increased the LDH levels significantly, but PMA caused a decrease in LDH levels. Interestingly, in case of LTA-induced inflammation, the lower dose of cathelicidin-2 did not influence the metabolic activity when compared to the LTA-only condition. In line with these results, in the absence of LTA exposure, cathelicidin-2 treatment showed an increase in LDH levels compared to the control, but with LTA supplementation, this elevation was not considerable. It has been found that cathelicidin-2 binds and neutralizes negatively-charged endotoxins like LPS and LTA, which plays a major role in its mechanism of action [29]. A model that might explain the data from the metabolic and LDH assays is that chicken cathelicidin-2 preferentially binds LTA; hence, no off-target cytotoxic effects could be observed in liver cell co-cultures when applying appropriate cathelicidin-2 concentrations. Excessive concentrations of the AMP, however, may result in unbound cathelicidin-2 and the development of off-target effects, as seen in the treatment conditions without LTA-induced inflammation.

Chicken cathelicidin-2 has been widely studied on various cell types in the previous years [19, 28, 30, 31], but there are no data about its effects on the hepatic cells of the chicken. In the present study, the concentration of four cytokines and chemokines was aimed to be assessed: CXCLi2 (the most similar chemokine in chickens to the human IL-8 [32]), IL-10, IFN-γ and M-CSF. These mediators are produced by a variety of cell types and have different and diversified mechanisms in influencing the inflammatory response. While CXCLi2 and IFN-γ can be considered as pro-inflammatory cytokines, the effect of M-CSF on the macrophages is much more complex. These molecules together with the anti-inflammatory IL-10 can provide us with an overview of the effects that chicken cathelicidin may have on the immune system in the liver [33–36].

The main role of IFN-γ is to activate immune cells, including macrophages, in order to stimulate the immune system to fight infections [35]. Upon activation by this cytokine, macrophages have been found to increase their phagocytic activity and their ROS and RNS production. IFN-γ has also been found to prime macrophages so that their responses to LPS challenges are heightened. This is most likely due to several pathways, for example, IFN-γ may serve to upregulate the expression of TLR4 receptor in macrophages [37]. It was found in our study that co-cultures treated with the higher dose of cathelicidin-2 produced increased levels of IFN-γ compared to the control. Similar results were found for a *Salmo salar* cathelicidin-derived peptide, applied to head kidney leukocytes in the absence of endotoxin-stimulated inflammation, resulting in increased expression of IFN-γ [38]. With regards to the cells exposed to only LTA, IFN-γ was produced at higher levels than the control, indicating that LTA had a pro-inflammatory effect in these cells. Cells treated with both LTA and 5 nmol/mL of cathelicidin-2 showed a decrease in IFN-γ levels when compared to those treated only with LTA. Based on these data, it can be stated that the lower dose of cathelicidin-2 could contribute to the restoration of the inflammatory homeostasis by successfully alleviating the LTA-triggered IFN-γ release, while the higher cathelicidin-2 concentration elicited pro-inflammatory activity. These results align with the findings of *Coorens et al*., where cathelicidin-2 proved to neutralize TLR2 and TLR4 activation by *Escherichia coli* [39]. Although cathelicidin-2 decreased the metabolic activity and increased the extracellular LDH levels, correlation between these parameters and the IFN- γ production could not be found, which indicates that the decreased IFN- γ levels were not a consequence of the reduced cell viability. PMA did not affect the IFN-γ levels, and cathelicidin-2 acted the same way when administered together with PMA as it did alone.

CXCLi2/IL-8 is a pro-inflammatory chemokine implicated with neutrophil (or in case of chickens, heterophil) infiltration into inflamed tissues [32, 35]. Neutrophil/heterophil infiltration is important for clearance of infection, but it should also be noted that these cells mount an aggressive response during inflammation that leads to tissue damage [40]. For this reason, IL-8 release is tightly controlled by anti-inflammatory molecules such as IL-10 [41].

According to our present results, an increase in CXCLi2 levels was found in the cell cultures treated with cathelicidin-2, providing more support for the theory that the role of cathelicidin-2 in the liver is immunomodulatory in nature rather than solely anti-inflammatory [19, 28]. This finding is in line with other studies found that human cathelicidins increased the expression and production of IL-8 [42, 43], and chicken cathelicidin-2 induced the transcription of CXCLi2 [19]. LTA or PMA did not cause significant changes in CXCLi2 levels compared to the control. The extracellular CXCLi2 concentration of cells exposed to LTA together with cathelicidin-2 was not different compared to those only treated with LTA, so the pro-inflammatory effect of the AMP was not observed when it was applied together with LTA. In contrast, combined cathelicidin-2 and PMA exposure caused similar elevation in CXCLi2 levels as the sole application of the AMP.

IL-10 acts indirectly to suppress the expression of pro-inflammatory genes and thereby keeps inflammatory responses regulated [34]. Cells treated with the higher dose of cathelicidin-2 showed a significant increase in IL-10 levels compared to the control, providing further evidence for the immunomodulatory function of cathelicidin-2 in the liver. Previous research has found that IL-10 initiates an important defense mechanism against inflammatory overshoot caused by IL-8, which explains the similarities between CXCLi2/IL-8 and IL-10 levels [41]. Increases in CXCLi2/IL-8 levels need to be offset with elevated IL-10 release to maintain inflammatory homeostasis in the liver cells. It should be noted that there were no significant differences between the cells exposed to LTA and the control. There was, however, a slight but significant decrease in IL-10 levels in the cells concomitantly exposed to LTA and the lower dose of cathelicidin-2. The data from the CXCLi2 and IL-10 assays coincide with the results from the cellular metabolism and LDH assays, altogether providing support for the theory that the binding of LTA to cathelicidin-2 results in the loss of its ability to induce cytokine release in chicken hepatocyte—non-parenchymal cell co-cultures.

The role of M-CSF in inflammation is especially complex. It stimulates the proliferation, differentiation, and activity of the mononuclear phagocyte cell lineage [44]. The biological functions of M-CSF are mediated by a tyrosine kinase receptor called CSF-1R that is expressed on all myeloid cells belonging to the mononuclear phagocytic lineage. Binding of M-CSF to its receptor results in dimerization and autophosphorylation of CSF-1R, triggering signalling cascades that contribute to cytoskeletal remodelling, increases in cell motility and the rate of protein synthesis and many other outcomes [36, 45]. This cytokine affects a range of macrophage-related immunological functions [44], including the release of IL-10 and IFN-γ, activation of anti-bacterial and anti-fungal activity, and increasing ROS production. M-CSF also plays a role in polarizing resident macrophages into anti-inflammatory type M2 macrophages [46]. Our results show that cathelicidin-2, alongside with LTA, decreased the extracellular concentration of M-CSF, while PMA elevated it. The M-CSF levels were further reduced following the treatment with the lower dose of cathelicidin-2 and LTA compared to the group receiving only LTA, and both concentrations of the peptide decreased the M-CSF levels of the PMA-exposed cells. These results suggest that cathelicidin-2 may suppress macrophage activation, also highlighting the multiplex immunomodulatory role of this AMP. However, it should be taken into consideration that beside the present in vitro data, further in vivo studies would be required to confirm this suggestion. Though LTA exposure evoked an increase in IFN-γ levels in the hepatocyte—non-parenchymal cell co-cultures, no changes were observed in CXCLi2 and IL-10 concentrations. Similarly, PMA only increased the production of M-CSF from the investigated cytokine/chemokine profile. These results indicate that both LTA and PMA had a pro-inflammatory action on the cells, but this was not reflected by all mediators assessed, probably due to the greatly varied effects of pro-inflammatory molecules in different species and tissue types [16, 43, 47]. The difference between the effect of LTA and PMA could be explained by the fact that PMA enters the pro-inflammatory pathway in a somewhat different manner, which could change the way it interferes with the production of cytokines [15].

Oxidative stress is associated with hepatic inflammatory processes because ROS and RNS are released from hepatocytes, Kupffer cells, and neutrophils in response to pathogen-associated molecular patterns (PAMPs) [48, 49]. Due to the immunomodulatory effects of AMPs, these peptides may play a role in influencing the redox balance as well. Some cathelicidins have been found to act against oxidative stress, but others, however, seem to increase ROS production of immune cells [50, 51]. Several studies found that cathelicidins had a protective effect against lipid peroxidation and the subsequent cell membrane damage [52, 53]. According to our results, cathelicidin-2 increased the production of H_2_O_2,_ but this effect was much less pronounced when applied together with LTA, possibly due to its suggested LTA-binding. Treatment with LTA also elevated the H_2_O_2_ production of the cultured hepatic cells, an effect which was counteracted by the lower dose of cathelicidin-2, indicating that neither the cathelicidin-2, nor the LTA could perform remarkable pro-oxidant action when applied together. However, these outcomes did not result in an increase in lipid peroxidation, as shown by the unchanged MDA concentrations, which indicates that the increased oxidative load did not lead to enhanced lipid peroxidation, so cathelicidin-2 did not contribute to oxidative damage of cell membranes. Moreover, the higher dose of the peptide decreased the MDA concentration, indicating a protective effect on the integrity of the membrane-forming phospholipids.

## Conclusion

In this study, the effects of cathelicidin-2 were investigated on the inflammatory and redox homeostasis in hepatocyte—non-parenchymal cell co-cultures of chicken origin. Based on our results, it can be stated that cathelicidin-2 plays a substantial role in modulating the hepatic immune response with a multifaceted mode of action. The higher concentration of the peptide was found to have more pronounced effects on metabolic activity, membrane integrity, and ROS production, indicating that using it in excessively high concentrations can lead to cell damage. However, the lower applied dose was not found to elicit any remarkable deteriorative action on cultured liver cells. Our findings give evidence that this molecule can possess anti-inflammatory properties, reflected by the alleviation of the LTA-triggered IFN-γ surge, and as a potent immunomodulator it can also stimulate pro-inflammatory CXCLi2 release balanced by enhanced anti-inflammatory IL-10 production. Further, the complex interplay of endotoxins and AMPs was highlighted as cathelicidin-2 showed less pronounced effects in the presence of LTA due to its binding capability, also neutralizing the endotoxin-associated inflammatory response. In conclusion, cathelicidin-2 seems to be a promising candidate for future immunomodulating drug development with an attempt to reduce the application of antibiotics, but further studies are required to investigate the inflammatory pathways affected by this peptide.

## Supporting information

S1 Table Dataset. (XLSX)

S2 Table Means and standard deviations of the treatment groups. (XLSX)
